# Epidemiology of Gunshot Wounds to the Nervous System Before and After the COVID-19 Pandemic: A 14-Year Single-Institution Retrospective Review

**DOI:** 10.7759/cureus.94032

**Published:** 2025-10-07

**Authors:** Nicholas P Derrico, Tyler Giles, Rebekah Kimball, Tyler Warner, Katherine E Baker, Evan Bowen, Thomas R Hemphill, John Wilkinson, Gregory R Vance, Zachary S Smalley

**Affiliations:** 1 Neurosurgery, University of Mississippi Medical Center, Jackson, USA

**Keywords:** covid-19, epidemiology children, gunshot wounds (gsw), neurotrauma, pediatric trauma

## Abstract

Firearm injuries continue to be an enduring and important United States public health issue, of which injuries to the nervous system are particularly disabling. The social dislocation caused by the COVID-19 pandemic has exacerbated this ongoing issue. Our group hypothesized that COVID-19 may have increased ballistic incidents and that this might have affected certain demographics more than others. The University of Mississippi Medical Center neurotrauma registry was searched for any gunshot wounds (GSWs) involving the nervous system and its coverings from Q1 2009 to Q2 2024. We defined Q2 2020 as the period when the COVID-19 pandemic began in the state of Mississippi. Our study at the state's only level I trauma center demonstrated that GSWs increased significantly from pre- to post-COVID-19, from an average of 16.80 GSWs per quarter pre-COVID-19 to 39.00 GSWs per quarter post-COVID-19. Adult GSWs accounted for 15.33 GSWs per quarter pre-COVID-19 and 31.56 GSWs per quarter post-COVID-19. Pediatric GSWs accounted for 1.42 GSWs per quarter pre-COVID-19 and 7.44 GSWs per quarter post-COVID-19. The increase was significant in both groups. The rate of change in GSWs per quarter, both pre- and post-COVID-19, was not significantly different in either group. The age distribution of pediatric GSWs demonstrated a similar bimodal distribution pre- and post-COVID-19, with approximately 2/3 of pediatric GSWs affecting those between 14 and 17 years of age. Our study reflects national trends in the distribution of GSWs and corroborates the finding that there has been a stepwise increase in pediatric GSWs since the onset of the COVID-19 pandemic. Our study suggests a need for increased attention to preventing gun-related injuries and demarcation of older adolescents (ages 14-17) as a high-risk group.

## Introduction

Firearm injuries remain a tenacious and critical United States public health concern. In 2019 and 2020, the National Emergency Department Sample database recorded 82,498 and 99,801 nonfatal firearm-related injuries, respectively. This represents an enormous cost to the US healthcare system, estimated at $493.2 billion in 2020 alone [1]. Multiple studies report that between 41.5% and 96% of this financial cost is borne by public funds either directly or indirectly [2,3]. Nationally, the number of firearm-related injuries is also increasing, and the COVID-19 pandemic is associated with an acceleration of this national trend [1,4-5]. At an urban level I trauma center, the two years post-lockdown showed significantly more firearm assaults, and the increase occurred at lockdown onset [4]. The pandemic was associated with a 34.3% increase in firearm-related nonfatal injuries and a 28.4% increase in firearm-related deaths [5]. The US Surgeon General, Dr. Vivek Murthy, ultimately declared firearm violence to be a public health crisis in 2024 [6].

Firearm injuries to the nervous system are particularly morbid. A study by Richmond et al. used US national databases to estimate the amount of lost life expectancy in survivors of gunshot wounds (GSWs) to the head and spine. Life expectancy for the entire US population decreases by 106.7 days due to firearm injuries to the brain and spine. In those rendered paraplegic, lost life expectancy ranges from 5.2 to 12.4 years for adults and 12.7 to 12.8 years for pediatric patients. In those who become ventilator-dependent, adults lose between 8.7 and 41.5 years of life expectancy, whereas children lose 43.6-44.4 years [7].

The state of Mississippi specifically has seen the highest age-adjusted death rate among all 50 states from 2020 to 2022. In 2022, Mississippi experienced 29.6 deaths per 100,000 for a total of 848 deaths [8]. The University of Mississippi Medical Center is the state’s only level I adult and pediatric trauma center. Given Mississippi's rate of GSWs, our group sought to better understand the demographics of this issue. Over approximately 14 years, the University of Mississippi Medical Center neurosurgical service evaluated 1,383 GSW patients with injuries to the head, spine, or peripheral nervous system. We report data from a level I trauma center, showcasing changes in GSW injuries over the past 14 years, with a focus on changes in event rates and patient characteristics since the start of the pandemic.

## Materials and methods

Study design and setting

The University of Mississippi Medical Center maintains a neurotrauma registry of patients evaluated by the neurosurgical service with a traumatic complaint. The registry is an IRB-approved retrospective study with no patient interaction or deviation from typical clinical care. The registry was queried for GSWs to the nervous system and its coverings from January 2009 to July 2024. Patients were included if their age could be determined and if they could be verified on chart review to have had a GSW to the nervous system and its coverings on that admission. Patients were excluded if their identity was unknown, and we could not verify their age. Adult (18 years of age and older) and pediatric patients were analyzed together and separately. We defined April 1, 2020, as the distinguishing date between pre- and post-COVID-19, as this was the date when Mississippi Governor Tate Reeves declared a statewide shelter-in-place order.

Data collection and statistical analysis

Registry query information and data from chart review were maintained in a secure, HIPAA-compliant database at the University of Mississippi Medical Center. The study was approved by the University of Mississippi Medical Center Institutional Review Board (approval number: UMMC-IRB-2023-329), and a waiver of informed consent was obtained for retrospective chart review.

Our data analysis plan was defined prior to the commencement of data collection. We performed Mann-Whitney U tests to compare the means of unpaired non-parametric data for adults and children in both pre- and post-COVID-19 cohorts. We also performed simple linear regressions to assess the rates of change pre- and post-COVID-19. Data analysis was performed through Excel (Microsoft Corp., Redmond, WA, USA) and Prism/GraphPad (GraphPad Software, LLC., Boston, MA, USA). We compared the proportion of each age group in the pediatric cohort using a chi-squared test (equivalent to a z-test for proportions) to determine if there were significant differences pre- and post-COVID-19. Finally, through another chi-squared test, we analyzed the distribution of GSW injuries by bodily location to compare the proportions of these injuries pre- and post-COVID-19.

## Results

Over approximately 14 years, the University of Mississippi Medical Center neurosurgical service evaluated 1,383 GSW patients with injuries to the head, spine, or peripheral nervous system. There were 1,151 adult patients and 227 pediatric patients. Five patients’ ages could not be identified and were thus excluded. Pre-COVID-19, 86.8% (646/744) of GSWs occurred in adults, while 79.7% (505/634) occurred in adults post-COVID-19. Pediatric patients accounted for 13.2% (98/744) of GSWs pre-COVID-19 and 20.3% (129/634) post-COVID-19. The average age for all patients was 31.6 ± 15.4 years pre-COVID-19 and 27.8 ± 13.2 years post-COVID-19. The average age of adults was 34.5 ± 14.5 years pre-COVID-19 and 31.5 ± 12.1 years post-COVID-19. Among pediatric patients, the average age was 12.9 ± 4.2 years pre-COVID-19 and 13.1 ± 4.7 years post-COVID-19. Approximately 85.3% (635/744) of all patients were male pre-COVID-19 compared to 83.9% (532/634) post-COVID-19. This predominantly male characteristic was maintained in the adult population (85.8% (554/646) pre-COVID-19 and 84.2% (425/505) post-COVID-19) and pediatric population (82.7% (81/98) pre-COVID-19 and 83.0% (107/129) post-COVID-19) (Table 1).

**Table 1:** Demographics of adult, pediatric, and all patients pre- and post-COVID-19. COVID-19: coronavirus disease 2019, PNS: peripheral nervous system

	Pre-COVID-19	Post-COVID-19
All	744	634
Male	635 (85.3)	532 (83.9)
Female	109 (14.7)	102 (16.1)
Average age	31.6 ± 15.4	27.8 ± 13.2
Head	376 (50.5)	277 (43.7)
Spine	289 (38.8)	313 (49.4)
PNS	92 (12.4)	45 (7.1)
Adult	646 (86.8)	505 (79.7)
Male	554 (85.8)	425 (84.2)
Female	92 (14.2)	80 (15.8)
Average age	34.5 ± 14.5	31.5 ± 12.1
Head	313 (48.5)	217 (43.0)
Spine	261 (40.4)	258 (51.1)
PNS	85 (13.2)	31 (6.1)
Pediatric	98 (13.2)	129 (20.3)
Male	81 (82.7)	107 (83.0)
Female	17 (17.3)	22 (17.0)
Average age	12.9 ± 4.2	13.1 ± 4.7
Head	63 (64.3)	60 (46.5)
Spine	28 (28.6)	55 (42.6)
PNS	7 (7.1)	14 (10.9)

**Figure 1:**
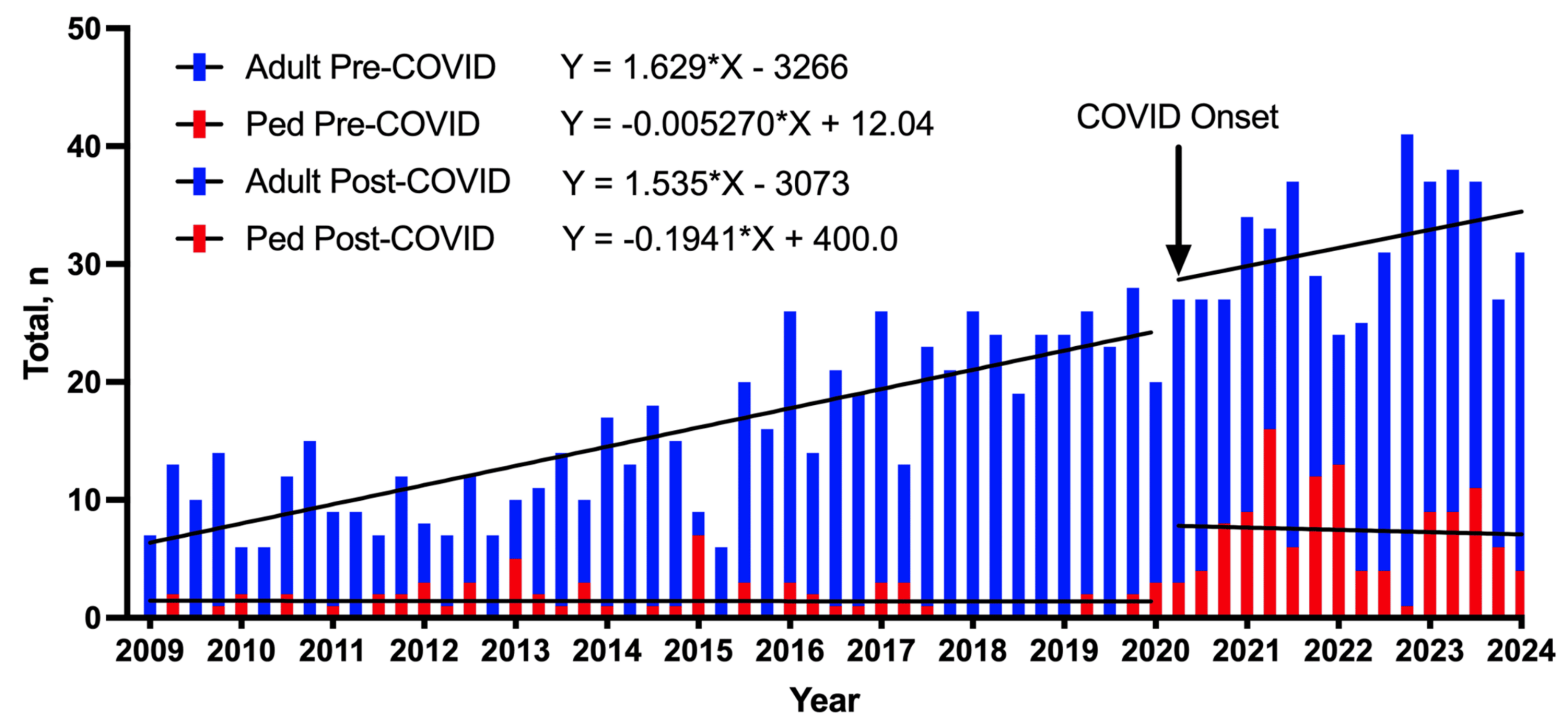
Overlapping bar chart of GSWs to the head, spine, or peripheral nerves for adult and pediatric populations from Q1 2009 to Q2 2024. Linear regression lines of best fit are included. GSWs: gunshot wounds

In total, there was an increase from an average of 16.80 GSWs pre-COVID-19 to 39.00 GSWs per quarter post-COVID-19. Linear regression analysis indicated that the rate of change for all GSWs was 1.62 pre-COVID-19 and 1.34 post-COVID-19. When adults and children were evaluated separately, there was an average of 15.33 adult GSWs per quarter compared to 31.56 in the post-COVID-19 quarters (Q2 2020 to Q2 2024), which was significantly different (p < 0.0001). In both pre- and post-COVID-19 periods, the average increase in adult GSWs per quarter was not significantly different, at 1.62 and 1.53, respectively. In the pediatric cohort, there was an average of 1.42 GSWs per quarter pre-COVID-19 versus 7.44 post-COVID-19, which was significantly different (p < 0.0001). The rate of change in GSWs per quarter, 0.00 pre-COVID-19 and -0.19 post-COVID-19, was not significantly different for the pediatric cohort (Figure 1).

**Figure 2:**
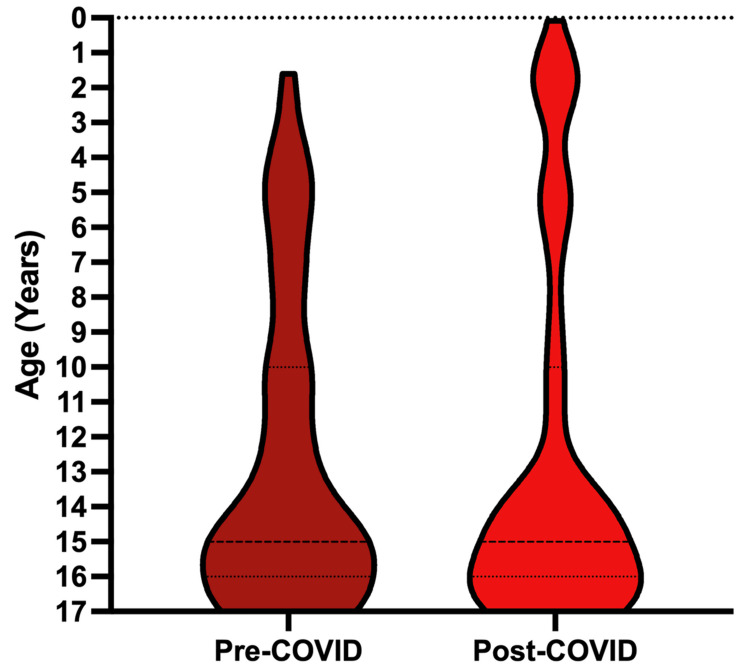
Age distribution of pediatric GSWs to the head, spine, or peripheral nerves, including patients from Q1 2009 to Q2 2024. GSWs: gunshot wounds

Among the pediatric patients, the distribution by age pre- and post-COVID-19 was quite similar (Figure 2). The proportion of each age group, ranging from those less than 1 to those aged 17, was not significantly different pre- and post-COVID-19 (p = 0.1101). Older adolescents remained the majority of pediatric GSW events, with children ages 14-17 accounting for 61.2% (60/98) of all pediatric GSWs pre-COVID-19 and 70.5% (91/129) post-COVID-19.

**Figure 3:**
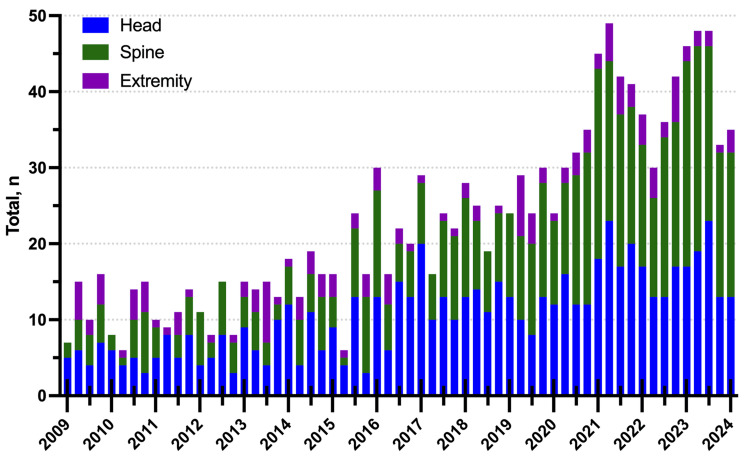
Stacked bar chart distribution of GSWs to the head, spine, and peripheral nerves in the entire cohort from Q1 2009 to Q2 2024. GSWs: gunshot wounds

The distribution of GSWs by location did not differ between pre- and post-COVID-19 periods. Pre-COVID-19, approximately 50.5% (376/744) of ballistic nervous system injuries involved the head, 38.8% (289/744) involved the spine, and 12.4% (92/744) involved the peripheral nerves. Within the adult population pre-COVID-19, 48.5% (313/646) of injuries implicated the head, 40.4% (261/646) implicated the spine, and 13.2% (85/646) implicated the peripheral nervous system. 13 adults in the pre-COVID-19 population were injured in two areas as opposed to one (e.g., head and spine). Within the pediatric population pre-COVID-19, 64.3% (63/98) of injuries involved the head, 28.6% (28/98) involved the spine, and 7.1% (7/98) involved the peripheral nervous system. Compared to post-COVID-19, there were 43.7% (277/634) firearm-related injuries to the head, 49.4% (313/634) to the spine, and 7.1% (45/634) to the peripheral nerves among all patients. For the adult population post-COVID-19, 43.0% (217/634) of injuries involved the head, 51.1% (258/634) involved the spine, and 6.1% (31/634) involved the peripheral nerves. One adult in the post-COVID-19 population was injured in both the spine and peripheral nerves and was the only patient in the post-COVID-19 population to have injuries in two areas. Finally, for the pediatric population post-COVID-19, 46.5% (60/129) of injuries involved the head, 42.6% (55/129) involved the spine, and 10.9% (14/129) involved the peripheral nervous system (Table 1). The proportion of GSWs to the head, spine, and extremity per quarter was not significantly different pre- and post-COVID-19 (p = 0.5357) (Figure 3).

## Discussion

Our results show that GSW injuries, particularly among children, increased dramatically over the study period. This not only concurs with a national trend but also mirrors the spike seen elsewhere post-COVID-19. The firearm homicide rate during the first year of the COVID-19 pandemic has been reported to increase between 28.4% [5] and nearly 35% [9]. The disproportionate increase in pediatric injuries has also been noted nationally. A retrospective cohort study of 49 tertiary care pediatric hospitals in the Pediatric Health Information System across the United States found a 52% increase in firearm-related injuries among children [10]. A separate retrospective study that reviewed data from the Pediatric Emergency Care Applied Research Network found that rates of visits for firearm injuries were significantly higher than expected among children 10-14 years old and 15-17 years old [11]. At a level I trauma center in Houston, they found that the total number of pediatric firearm-related injury cases increased during the pandemic over the same period of time. These injuries primarily affected males aged 14 to 17 [12]. Our results concur with this trend observed at both the national and individual hospital levels. As hypothesized, GSWs not only increased but also affected certain groups more severely. This work identifies the pediatric population, particularly older adolescents, as a high-risk group and part of an increase in GSWs in association with the COVID-19 pandemic.

Possible etiologies for this predominantly pediatric increase in GSW injuries are many. Societal stressors increased, and that has been thought to contribute to the change in trauma patterns observed during the pandemic, with fewer vehicular accidents and more home-based injuries and non-accidental trauma [13]. This trend has also been demonstrated to increase the proportion of violent as opposed to accidental sources of trauma, with nationwide increases in pediatric stabbing (3.3%, up from 2.8%) and GSWs (5.5%, up from 4.0%) as a proportion of total trauma presentations [14]. Access to firearms, with children spending so much more time at home, is another concern. Survey data suggest that in the wake of the COVID-19 pandemic, nearly 40% of survey respondents of new firearm purchases reported non-secure firearm storage [15]. Additionally, the rates of unintentional pediatric shootings have been noted to be 24-72% lower in states with mandated secure storage laws [16]. Mississippi is not one of these states.

Interestingly, in the pediatric cohort, our study reveals that the increase in events was not gradual over time, but rather a fairly abrupt rise in GSWs per quarter at the onset of the COVID-19 pandemic. This increase was maintained into 2023, after much of the social dislocation associated with the pandemic had returned toward normal. In adults, conversely, we observe a fairly steady increase of approximately 1.5 additional GSWs per quarter, both before and after the onset of the COVID-19 pandemic. While it has been reported elsewhere that there is an increase in GSWs overall and in pediatric populations after the pandemic, the shape of this increase, which is gradual in adults and abrupt in children, has not been previously described.

Limitations

Our study is limited primarily by being a retrospective review of a single institution's experience. It is also associational and unable to attribute causation to its findings. Further research is needed to clarify the attribution of these associations.

## Conclusions

Our 14-year study at a single level I trauma center demonstrated the national trend that GSWs to the nervous system increased from pre- to post-COVID-19. Pediatric GSWs particularly increased after the pandemic, concurring with the national trend. The pattern of increase in adult GSWs was gradual, whereas the increase in pediatric GSWs was abrupt with the onset of the pandemic. This increase could be due to a multitude of factors, including shifts in societal stressors and increased access to firearms at home. Our study supports the growing body of evidence of increased firearm-related injuries in the wake of the COVID-19 pandemic, both indicating a need for increased attention to this issue and to identify older adolescents as a high-risk group.

## References

[1] Miller GF, Barnett SB, Florence CS, McDavid Harrison K, Dahlberg LL, Mercy JA (2024). Costs of fatal and nonfatal firearm injuries in the U.S., 2019 and 2020. Am J Prev Med.

[2] Ordog GJ, Wasserberger J, Ackroyd G (1995). Hospital costs of firearm injuries. J Trauma.

[3] Spitzer SA, Forrester JD, Tennakoon L, Spain DA, Weiser TG (2022). A decade of hospital costs for firearm injuries in the United States by region, 2005-2015: government healthcare costs and firearm policies. Trauma Surg Acute Care Open.

[4] Krzyzaniak A, Carroll AN, Rooney AS, Calvo RY, Bansal V, Sise MJ (2023). Firearm assaults in communities: the impact of COVID-19 lockdown. Am Surg.

[5] Sun S, Cao W, Ge Y, Siegel M, Wellenius GA (2022). Analysis of firearm violence during the COVID-19 pandemic in the US. JAMA Netw Open.

[6] Office of the Surgeon General. (2024, June 25 (2024). U.S. surgeon general issues advisory on the public health crisis of firearm violence in the United States. U.S. Surgeon General Issues Advisory on the Public Health Crisis of firearm violence in the United States.

[7] Richmond TS, Lemaire J (2008). Years of life lost because of gunshot injury to the brain and spinal cord. Am J Phys Med Rehabil.

[8] Centers for Disease Control and Prevention. (2022, March 1 (2024). Firearm mortality. https://www.cdc.gov/nchs/state-stats/deaths/firearms.html.

[9] Centers for Disease Control and Prevention. (2022b, May 12 (2024). Vital signs: changes in firearm homicide and suicide rates — United States, 2019-2020. https://www.cdc.gov/mmwr/volumes/71/wr/mm7119e1.htm.

[10] Iantorno SE, Swendiman RA, Bucher BT, Russell KW (2023). Surge in pediatric firearm injuries presenting to us children’s hospitals during the COVID-19 pandemic. JAMA Pediatr.

[11] Hoffmann JA, Carter CP, Olsen CS (2023). Pediatric firearm injury emergency department visits from 2017 to 2022: a multicenter study. Pediatrics.

[12] Orantes C, Chan HK, Walter D, Chavez S, Ugalde IT (2023). Pediatric firearm injury epidemiology at a level 1 trauma center from 2019 to 2021: including time of the COVID-19 pandemic. Inj Epidemiol.

[13] Lee YY, Loo LM, Oh E, Ang IW, Menon RK (2024). Pediatric trauma during the COVID-19 lockdown: caregiver abuse and self-harm in a vulnerable population. Pediatr Surg Int.

[14] Garcia L, de Virgilio C, Nahmias J, Keeley JA, Grigorian A (2024). The relationship between the COVID-19 pandemic and pediatric trauma. J Surg Res.

[15] Lyons VH, Haviland MJ, Azrael D (2021). Firearm purchasing and storage during the COVID-19 pandemic. Inj Prev.

[16] Cannon AD, Reese K, Tetens P, Fingar KR (2023). Preventable tragedies: findings from the #NotAnAccident index of unintentional shootings by children. Inj Epidemiol.

